# Supplementary material for: Neural dynamics between anterior insular cortex and right supramarginal gyrus dissociate genuine affect sharing from perceptual saliency of pretended pain
Source: eLife. 2021 Aug 19;10:e69994. doi: 10.7554/eLife.69994 (PMC8443248; doi:10.7554/eLife.69994)
Supplement: Supplementary file 1. — Smaller AIC/BIC indicates better model fit. Results showed that M1 (without interaction; highlighted with underlining) was the best fitting model for both genuine pain and pretended pain. [file elife-69994-supp1.docx]

**Supplementary Table 1.** Model comparison of linear regression models with three behavioral ratings (independent variables) and the inhibitory effect (dependent variable) for genuine pain and pretended pain. Smaller AIC/BIC indicates better model fit. Results showed that M1 (without interaction; highlighted with underlining) was the best fitting model for both genuine pain and pretended pain.

| Regression Model | AIC | BIC |
| --- | --- | --- |
| Genuine pain |  |  |
| M1 (expression + feeling + unpleasantness) | 10.069 | 18.757 |
| M2 (expression * feeling + unpleasantness) | 11.342 | 21.768 |
| M3 (expression + feeling * unpleasantness) | 11.195 | 21.621 |
| M4 (expression * unpleasantness + feeling) | 11.126 | 21.552 |
| M5 (expression * feeling * unpleasantness) | 16.153 | 31.792 |
| Pretended pain |  |  |
| M1 (expression + feeling + unpleasantness) | 51.041 | 59.919 |
| M2 (expression * feeling + unpleasantness) | 51.230 | 61.467 |
| M3 (expression + feeling * unpleasantness) | 52.643 | 63.069 |
| M4 (expression * unpleasantness + feeling) | 52.584 | 63.010 |
| M5 (expression * feeling * unpleasantness) | 55.200 | 70.839 |
