# Supplementary material for: Neural dynamics between anterior insular cortex and right supramarginal gyrus dissociate genuine affect sharing from perceptual saliency of pretended pain
Source: eLife. 2021 Aug 19;10:e69994. doi: 10.7554/eLife.69994 (PMC8443248; doi:10.7554/eLife.69994)
Supplement: Transparent reporting form [file elife-69994-transrepform1.pdf]

eLIFE
1st Floor
24 Hills Road
Cambridge CB2 1JP, UK
P 01223 855340
W elifesciences.org
T @elife

eLife's transparent reporting form

We encourage authors to provide detailed information *within their submission* to facilitate the interpretation and replication of experiments. Authors can upload supporting documentation to indicate the use of appropriate reporting guidelines for health-related research (see EQUATOR Network), life science research (see the BioSharing Information Resource), or the ARRIVE guidelines for reporting work involving animal research. Where applicable, authors should refer to any relevant reporting standards documents in this form.

If you have any questions, please consult our Journal Policies and/or contact us: editorial@elifesciences.org.

Sample-size estimation

- You should state whether an appropriate sample size was computed when the study was being designed
- You should state the statistical method of sample size computation and any required assumptions
- If no explicit power analysis was used, you should describe how you decided what sample (replicate) size (number) to use

Please outline where this information can be found within the submission (e.g., sections or figure legends), or explain why this information doesn't apply to your submission:

This sample size was determined on a priori power analysis in Gpower 3.1, with a medium effect size of Cohen's d = 0.5. This information can be found in the section of Materials and Methods.

Replicates

- You should report how often each experiment was performed
- You should include a definition of biological versus technical replication
- The data obtained should be provided and sufficient information should be provided to indicate the number of independent biological and/or technical replicates
- If you encountered any outliers, you should describe how these were handled
- Criteria for exclusion/inclusion of data should be clearly stated
- High-throughput sequence data should be uploaded before submission, with a private link for reviewers provided (these are available from both GEO and ArrayExpress)

Please outline where this information can be found within the submission (e.g., sections or figure legends), or explain why this information doesn't apply to your submission:

The video clips used in this study were validated and a pilot study was performed to ensure the experimental effect. The number of participants and the criteria for exclusion/inclusion and outliers are provided. All this information in detail can be found in the section of Materials and Methods. Raw functional imaging and behavioral data are deposited at https://doi.org/10.5281/zenodo.4783235. Processed behavioral data and individual DCM parameters are accessible at https://github.com/Yili-Zhao/Genuine_pretended-pain-task.git. Unthresholded statistical maps are available at https://identifiers.org/neurovault.collection:9949.

eLife Sciences Publications, Ltd is a limited liability non-profit non-stock corporation incorporated in the State of Delaware, USA, with company number 5030732, and is registered in the UK with company number FC030576 and branch number BR015634 at the address 1st Floor, 24 Hills Road, Cambridge CB2 1JP | August 2014

Statistical reporting

- Statistical analysis methods should be described and justified
- Raw data should be presented in figures whenever informative to do so (typically when N per group is less than 10)
- For each experiment, you should identify the statistical tests used, exact values of N, definitions of center, methods of multiple test correction, and dispersion and precision measures (e.g., mean, median, SD, SEM, confidence intervals; and, for the major substantive results, a measure of effect size (e.g., Pearson's r, Cohen's d)
- Report exact p-values wherever possible alongside the summary statistics and 95% confidence intervals. These should be reported for all key questions and not only when the p-value is less than 0.05.

Please outline where this information can be found within the submission (e.g., sections or figure legends), or explain why this information doesn't apply to your submission:

All the statistical details required above can be found in sections of Materials and Methods and Results; except for the second point which does not apply to this submission as N in the group is > 40.

(For large datasets, or papers with a very large number of statistical tests, you may upload a single table file with tests, Ns, etc., with reference to sections in the manuscript.)

Group allocation

- Indicate how samples were allocated into experimental groups (in the case of clinical studies, please specify allocation to treatment method); if randomization was used, please also state if restricted randomization was applied
- Indicate if masking was used during group allocation, data collection and/or data analysis

Please outline where this information can be found within the submission (e.g., sections or figure legends), or explain why this information doesn't apply to your submission:

As a within-subject design, all participants were allocated to the experimental group. Particularly, we counterbalanced the demonstrators appearing in the genuine and pretended conditions across participants in a random order, thus controlling for differences in behavioral and brain response that could be explained by differences between the stimulus sets. This information can be found in the section of Materials and Methods.<br>Masking does not apply to this study as for a within-subject design.

Additional data files ("source data")

- We encourage you to upload relevant additional data files, such as numerical data that are represented as a graph in a figure, or as a summary table
- Where provided, these should be in the most useful format, and they can be uploaded as "Source data" files linked to a main figure or table
- Include model definition files including the full list of parameters used
- Include code used for data analysis (e.g., R, MatLab)
- Avoid stating that data files are "available upon request"

Please indicate the figures or tables for which source data files have been provided:

Raw functional imaging and behavioral data are deposited at https://doi.org/10.5281/zenodo.4783235. Processed behavioral data and individual DCM parameters are accessible at https://github.com/Yili-Zhao/Genuine_pretended-pain-task.git. Unthresholded statistical maps are available at https://identifiers.org/neurovault.collection:9949. Main clusters of mass-univariate function segregation analyses are completely reported in Table 1.
